# Molecular Epidemiological Characterisation of ESBL- and Plasmid-Mediated AmpC-Producing *Escherichia coli* and *Klebsiella pneumoniae* at Kamuzu Central Hospital, Lilongwe, Malawi

**DOI:** 10.3390/tropicalmed7090245

**Published:** 2022-09-14

**Authors:** Faheema Ebrahim Choonara, Bjørg Christina Haldorsen, Jessin Janice, Joshua Mbanga, Isaac Ndhlovu, Osborne Saulosi, Tarsizio Maida, Fanuel Lampiao, Gunnar Skov Simonsen, Sabiha Yusuf Essack, Arnfinn Sundsfjord

**Affiliations:** 1 Antimicrobial Research Unit, College of Health Sciences, University of KwaZulu-Natal, Private Bag X54001, Durban 4041, South Africa; 2 Microbiology Laboratory, Kamuzu Central Hospital, Lilongwe P.O. Box 149, Malawi; 3 Africa Centre of Excellence in Public Health and Herbal Medicine, College of Medicine, University of Malawi, Private Bag 360, Blantyre 3, Malawi; 4 Norwegian National Advisory Unit on Detection of Antimicrobial Resistance (K-res), Department of Microbiology and Infection Control, University Hospital of North Norway, 9038 Tromsø, Norway; 5 Department of Applied Biology and Biochemistry, National University of Science and Technology, Corner Cecil & Gwanda Road, Private Bag 939, Bulwayo P.O. Box AC939, Zimbabwe; 6 Research Group for Host-Microbe Interactions, Department of Medical Biology, Faculty of Health Sciences, UiT The Arctic University of Norway, 9037 Tromsø, Norway

**Keywords:** *Escherichia coli*, *Klebsiella pneumoniae*, bla_CTX-M-15_, bla_CMY_, clonal spread

## Abstract

The global rise in infections caused by multidrug resistant (MDR) Enterobacterales poses a public health problem. We have performed a molecular epidemiological characterisation of representative plasmid-mediated AmpC (pAmpC) and ESBL-positive clinical isolates of *Escherichia coli* (n = 38) and *Klebsiella pneumoniae* (n = 17) from a tertiary hospital in Malawi collected in 2017. *Bla*_CTX-M-15_ was the most prevalent ESBL-determinant in *E. coli* (n = 30/38) and *K. pneumoniae* (n = 17/17), whereas *bla*_CMY-2_ was detected in nearly all AmpC-phenotype *E. coli* (n = 15/17). Whole genome sequencing revealed dominant globally disseminated *E. coli* sequence types (STs); ST410 (n = 16), ST131 (n = 7), and ST617 (n = 6). The ST distribution in *K. pneumoniae* was more diverse but included ST101 (n = 2), ST14 (n = 2), and ST340 (n = 2), all considered high-risk MDR clones. The isolates expressed an MDR profile, including resistance against commonly used antibiotics, such as fluoroquinolones, aminoglycosides, and/or trimethoprim-sulfamethoxazole, and harboured corresponding resistance determinants. Clonal analyses of the major STs of *E. coli* revealed closely related genetic clusters within ST410, ST131, and ST617 supporting within-hospital transmission between patients and/or via a common reservoir. The overall findings add to the limited knowledge on the molecular epidemiology of MDR *E. coli* and *K. pneumoniae* in Malawi and may help health policy makers to identify areas to target when addressing this major threat of antibiotic resistance.

## 1. Introduction

The global rise in infections caused by multidrug resistant (MDR) Enterobacterales poses a public health threat due to delayed effective therapy and poorer clinical outcome [1,2]. The problem is dominated by extended-spectrum β-lactamase (ESBL) and plasmid-mediated AmpC (pAmpC) producing *Escherichia coli* and *Klebsiella pneumoniae*, and the dissemination of their MDR clonal lineages [3,4,5]. It is important to detect such isolates to optimize patient treatment and implement infection-control measures.

ESBLs and pAmpCs differ in biochemical characteristics, which can be used for phenotypic detection. While both enzyme groups, in general, hydrolyse penicillins, narrow- and extended-spectrum cephalosporins, and monobactams, only pAmpCs hydrolyse cephamycins and are inhibited by fourth-generation cephalosporins such as cefepime and cefpirome [6]. In contrast to ESBLs, pAmpCs are poorly inhibited by the traditional β-lactamase inhibitors such as clavulanic acid and tazobactam [7].

Differences in amino acid sequence give rise to different families of pAmpCs [6]. CMY-2 enzymes are the most common pAmpC variants worldwide [6]. The distribution of pAmpC in clinical isolates of Enterobacterales is underexplored in sub-Saharan Africa. However, a faecal carriage study of university students in Mozambique detected pAmpC alone in 11% and in co-existence with ESBL (CTX-M) in 36% of *E. coli* and *K. pneumoniae* isolates resistant to third-generation cephalosporins [8].

ESBLs are more prevalent than pAmpCs in clinical isolates of Enterobacterales worldwide [2,6,9,10,11]. CTX-Ms are the most widespread ESBLs followed by subtypes of TEM and SHV, with CTX-M-15 being the most dominant allelic variant in Africa [9,10]. A worldwide increase in community-acquired CTX-M type ESBL-producing *E. coli* has been observed in the last decade, with developing countries being affected the most [12].

A systematic review of ESBL-producing Enterobacterales in Africa revealed a diverse prevalence of ESBL-producing Enterobacterales dependent upon geographical locations and populations [13]. The emergence of ESBL-producing *E. coli* and *K. pneumoniae* and the association with MDR clonal lineages has recently been described in Malawi [14,15,16]

It is important to distinguish between pAmpC- from ESBL-producing Enterobacterales due to differences in β-lactam susceptibility and propensity for nosocomial dissemination [6]. Moreover, pAmpC can mask phenotypic confirmation of ESBLs resulting in false negative ESBL tests [17,18]. In a prospective observational study in 2017 at Kamuzu Central Hospital (KCH), a referral hospital in Lilongwe, Malawi, we phenotypically identified a high prevalence of ESBL- and AmpC-producing Enterobacterales [19]. The aim of this study was to perform a molecular epidemiological characterisation of a representative selection of ESBL- and/or AmpC-phenotype positive Enterobacterales.

## 2. Materials and Methods

The bacterial strains were collected from June to December 2017 at KCH, a governmental referral hospital for the central region of Malawi serving a community of 6 million people with approximately 750 beds. KCH has four major hospital departments: medical, obstetrics and gynaecology, surgical and paediatrics. Microbiology specimens were collected from hospitalised adult patients (>18 years) suspected to have a clinical infection. Specimen types included urine, blood cultures, cerebrospinal fluid (CSF), other sterile fluids, and pus. The clinical staff collected the samples based on the clinical diagnosis of infection made by a physician. Data collection including identification of bacterial isolates and antimicrobial susceptibility testing (AST) was performed as previously described [19].

Briefly, Gram negative isolates were identified using analytical profile index (API) 20E and 20NE systems (BioMerieux, Durham, NC, USA) and subjected to antimicrobial susceptibility testing (AST). The disk diffusion method as per the EUCAST guidelines was used and zone diameters were interpreted using the EUCAST clinical breakpoints version 4 (http://www.eucast.org/clinical_breakpoints/) (accessed on 28 July 2021). Detection of ESBL- and/or AmpC-phenotypes was performed using the combination disk test with clavulanic acid and cloxacillin, respectively, on isolates with reduced susceptibility to cefotaxime and/or ceftazidime [19].

Phenotypic quality control (QC) strains included *E. coli* CCUG 58543, ESBL-positive *K. pneumoniae* NCTC 13368/ATCC 700603, as well as *Escherichia coli* ATCC 25922. Clinical isolates were stored at −80 °C in tryptone soy broth with 10% glycerol until shipping to Norwegian Advisory Unit on Detection of Antimicrobial Resistance, Tromsø, Norway, for further molecular testing.

### 2.1. Selection of ESBL- and/or AmpC-Phenotype Positive Isolates for Genetic Characterisation

Isolates for genetic characterisation were selected from clinical isolates that were identified and screened for ESBL- and/or AmpC-phenotype from our previous study [19]. These isolates comprised a total of 174 clinical isolates of Enterobacterales as previously described [19], which included a total number of *E. coli* (n = 92), *Enterobacter cloacae* (n = 13), *K. pneumoniae* (n = 29), *Proteus mirabilis* (n = 33), and *Salmonella* spp. (n = 7). ESBL-production was phenotypically confirmed in only 86/174 (49%) of the Enterobacterales isolates as described above, which included *E. coli* (n = 49), *K. pneumoniae* (n = 20), *E. cloacae* (n = 6), and *P. mirabilis* (n = 11). Of those, 49 isolates also expressed an AmpC-phenotype. A separate AmpC-phenotype was not observed [19]

All those 86 isolates that had reduced susceptibility to cefotaxime and/or ceftazidime and positive for ESBL and/or pAmpC by combination disk test with clavulanic acid and cloxacillin, respectively, as previously described were shipped to the Norwegian Advisory Unit on Detection of Antimicrobial Resistance, Tromsø, Norway, for molecular analyses. Upon arrival, the isolates were re-cultured on lactose agar, re-identified using MALDI-TOF (Bruker Daltonik, Bremen, Germany), as well as retested for an ESBL- or AmpC-phenotype using the ROSCO ESBL and AmpC confirmation kits (ROSCO, Taastrup, Denmark), respectively.

### 2.2. PCR Analysis for ESBL and pAmpC β-Lactamase Genes

DNA was extracted using the bioMerieux NucliSENS-easyMAG (bioMerieux, Marcy l’Étoile, France) and subjected to real-time PCR for the detection of CTX-M type ESBL and pAmpC using the Applied Biosystems 7500 Fast Real-Time PCR system (Applied Biosystem Inc., Foster City, CA, USA). Genes encoding CTX-M group 1/2/9, and CTX-M (consensus) as well as CIT, CMY, FOX, MOX, DHA, ACC, and EBC type pAmpC were detected as described in [20,21].

PCR QC strains included: For CTX-M RT-PCR; A2-23 *K. pneumoniae* (CTX-M gr1), A2-39 *E. coli* (CTX-M gr.2), A2-37 *K. pneumoniae* (CTX-M gr.9), A2-38 *K. pneumoniae* (CTX-M gr.9), and *E.coli* ATCC 25922. For the pAmpC RT-PCR; A2-57 *Citrobacter freundii* (CIT), A2-20 *P. stuartii* (CMY), A2-24 *K. pneumoniae* (CMY), A2-59 *K. oxytoca* (FOX), A4-27 *E. coli* (MOX), A2-61 *Hafnia alvei* (DHA), A2-60 *Morganella morganii* (ACC), A2-58 *Enterobacter* sp. (EBC), and ATCC 25922 *E. coli*. The A-number strains are all whole-genome-sequenced internal reference strains at the reference laboratory.

### 2.3. Whole Genome Sequencing (WGS) and Bioinformatics Analysis

Whole genome sequencing was performed using the MiSeq platform (Illumina, San Diego, CA, USA) according to the manufacturer’s instructions. Briefly, genomic DNA was purified using the EZ1 DNA Tissue kit (Qiagen, Hilden, Germany). DNA libraries were prepared using Nextera/Nextera XT kits (Illumina) followed by paired-end sequencing. Contigs were assembled using SPAdes v3.13.0 (St. Petersburg State University, St Petersburg, Russia). The quality control criteria included: (i) a minimum of 40x coverage, (ii) genomic length not lower than 95% of the smallest and not exceeding 105% of the largest closed species related genome on NCBI, and (iii) the total number of contigs below 400. The presence of resistance genes/mutations was determined using Abricate 0.9.8 using NCBI’s Bacterial Antimicrobial Resistance Reference Gene Database (PRJNA313047) as the reference and STs were determined from WGS data using MLST 2.16.2 database hosted by the Centre for Genomic Epidemiology (CGE) (http://cge.cbs.dtu.dk/services/MLST/) (accessed on 1 August 2021). Acquired antimicrobial resistance genes and chromosomal point mutations including the DNA gyrase *gyr*A, *parC* and *parE* genes (quinolone resistance) were annotated using ResFinder 4.1 (https://cge.cbs.dtu.dk/services/ResFinder/) (accessed on 4 August 2021). Plasmid replicon were identified using PlasmidFinder 2.1 on the CGE website (https://cge.cbs.dtu.dk/services/PlasmidFinder/) (accessed on 4 August 2021). The sequences have been deposited at GenBank under the Bioproject number PRJNA746135.

### 2.4. Analysis of Clonal Relatedness

To further distinguish *E. coli* strains within the same sequence type (ST), the SeqSphere+ software (Ridom, Münster, Germany) was used. The *E. coli* cgMLST scheme with reference K12 (NC_00913.3) with the core genome consisting of 2513 alleles was used to examine clonal relatedness among our *E. coli* genomes with a cluster distance threshold of ≤10 allelic differences.

## 3. Results

Only 60/86 (70%) of the shipped isolates were available in pure culture for phenotypic confirmation and molecular characterisation due to failure of growth upon arrival in the reference laboratory, mislabelling, or mixed culture: *E. coli* (n = 38), *K. pneumoniae* (n = 17), *P. mirabilis* (n = 4), and *E. cloacae* (n = 1). All 60 isolates were reconfirmed as ESBL positive. The AmpC phenotype was reconfirmed in 21/60 (35%) isolates: *E. coli* (n = 17), *P. mirabilis* (n = 3), and *E. cloacae* (n = 1). Due to low numbers of other species, we focused only on the molecular characterization of *E. coli* (n = 38) and *K. pneumoniae* (n = 17) in the following.

### 3.1. PCR Analysis for Genes Encoding CTX-M and pAmpC β-Lactamases

The results are summarized in Table 1 (*E. coli*) and Table 2 (*K. pneumoniae*). Briefly, *bla*_CTX-M Group 1_ was present in *E. coli* (n = 30/38) and *K. pneumoniae* (n = 17/17), while *bla*_CTX-M group 9_ was found in *E. coli* (n = 7). Thus, only one *E. coli* isolate was negative for *bla*_CTX-M_. The pAmpC-PCR was positive in nearly all AmpC-phenotype *E. coli* (n = 15/17), all *bla*_CMY_, indicating hyperproduction of the chromosomal AmpC-encoding gene in the two isolates which were negative pAmpC. One of the pAmpC-PCR negative isolates was also negative for *bla*_CTX-M_.

### 3.2. Whole Genome Sequencing (WGS)

#### *E. coli* ST-Profile and AMR-Determinants

All *bla*_CTX-M_ -positive *E. coli* (n = 37) were subjected to phylogenetic analyses, potential clonal relatedness, and to verify PCR findings. Detailed information on the individual isolates is given in Supplementary Table S1 (ST1). Table 1 summarizes the ST profiles and distribution of resistance determinants to clinically important classes of antibiotics for which we had phenotypic AST data: β-lactams, fluoroquinolones (FQ), trimethoprim (TMP), sulphonamides (SUL), and aminoglycosides (AG).

ST410 (n = 16), ST131 (n = 7), and ST 617 (n = 6) were the dominant STs among the eleven different STs. Notably, all pAmpC-positive (*bla*_CMY-2_) isolates (n = 15) were of ST410, and most *bla*_CTX-M Group 9_ (*bla*_CTX-M-27_) positive isolates were of ST131. ST410 isolates were prevalent in specimens from the surgical department (n = 11) and pus specimens (n = 10).

The presence of *bla*_CTX-M Group 1 and 9_ as well as *bla*_CMY_ as determined by PCR, was confirmed by WGS. *Bla*_CTX-M-15_ (n = 30) and *bla*_CTX-M-27_ (n = 6) were the dominant allelic variants in *bla*_CTX-M Group 1_ and *bla*_CTX-M Group 9_ positive isolates, respectively. All 37/37 (100%) isolates were resistant to cefotaxime, ceftazidime, and cefuroxime. All *bla*_CMY-2_ isolates were also resistant to cefoxitin. Other major β-lactamase encoding genes included *bla*_OXA-1_ (n = 23) and *bla*_TEM1-B_ (n = 32).

The dominant FQ-resistance determinants included *aac (6’)-Ib-cr* (n = 25). Chromosomal mutations affecting the DNA gyrase (n = 32) comprising *gyr*A (S83L); n = 32/37, *gyr*A (D87N); n = 29/37, *gyr*A (S83A); n = 1/37, and DNA topoisomerase IV (*par*C; n = 29, *par*E; n = 31) comprising *par*C (S80I); n = 29/37, *par*C (E84G); n = 1/37, *par*C (E84V); n = 3/37, and *par*E (E460D); n = 1/37; *par*E (I355T); n = 1/37, *par*E (1529L); n = 5/37, *par*E (S458A); n = 25/37 were also evident. Only two isolates did not contain any quinolone-resistance determinants. *Qnr*-resistance determinants were not detected in *E. coli*. We did not observe any inconsistencies between the genotypic presence of quinolone resistance and susceptibility to ciprofloxacin (Table S1).

TMP-resistance determinants included *dfr_A1_*, *dfr*_A8_, *dfr*_A12_, *dfr*_A14_, and *dfr*_A17_, of which *dfr*_A17_ (n = 31) was the dominant subtype. SUL-resistance genes were of subtypes *sul*_1_ and *sul*_2_ of which *sul*_2_ (n = 35) was dominant. Nearly all *E. coli* isolates (n = 36) contained at least one trimethoprim and one sulphonamide-resistance determinant and were associated with phenotypic resistance to trimethoprim-sulfamethoxazole (TMP-SUL). All ST410 and ST131 isolates were *dfr*_A17_ and *sul*_1/2_ positive.

The dominant AG-resistance determinants were *aph (6)-Id* (n = 35), *aph (3″)-Ib* (n = 33), *aadA5* (n = 31), and *aac (3)-IId* (n = 23), all of which were predominant in ST410. All isolates of *E. coli* contained at least one resistance determinant encoding AG-modifying enzymes with different substrate profiles. Gentamicin resistance was expressed in 28/37 (76%) of the *E. coli* isolates. Due to the current complexities in AG-resistance determinants and some inconsistencies in published substrate profiles, we did not perform any additional comparison of AG geno- and phenotype.

### 3.3. Plasmid Incompatibility Groups

A total of 17 incompatibility groups were identified among the *E. coli* isolates (ST1). IncFIB (n = 37/37; 100%), IncFIA (n = 34/37; 92%), and IncFII (n = 33/38; 89%) were most common. We did not perform any analyses of the association between Inc-groups and pheno- or genotypic characteristics.

### 3.4. Clonal Relatedness in the Major STs of E. coli

**Figure 1:**
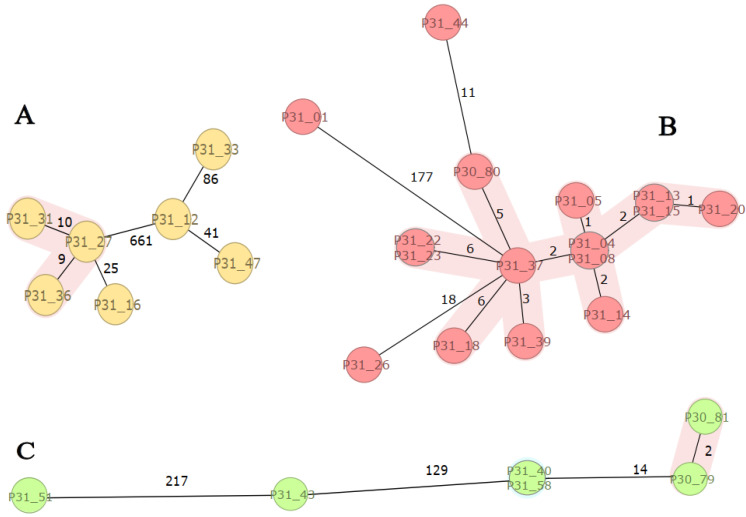
SeqSphere analysis of *E. coli* high-risk clones: ST131, ST410, and ST617. Figure Legend: Minimum spanning three based on cgMLST analysis of ESBL-producing *E. coli* ST131 ((**A**); n = 7), ST410 ((**B**); n = 16), and ST617 ((**C**); n = 6) isolates. The isolates are coloured by sequence type (ST) and numbered according to Supplementary Table S1. Clusters are coloured in pink and related to the cluster distance threshold of ≤10 allele differences as given in numbers along the lines between individual isolates.

SeqSphere-analyses of the major STs of *E. coli* (ST131, ST410, and ST617) revealed a close genetic relationship between isolates (clusters) within each ST (Figure 1). ST131 isolates (n = 7) were genetically diverse with a clonal cluster of three isolates (P31-27, -31, -36). Most of the ST410 isolates (n = 16) clustered except three (P31-01, -26, and -44), whereas two ST617 clusters, P30-79 and -81 as well as P31-40 and -58, were observed. The results are consistent with the fact that each cluster has a common origin in the recent past indicating transmissions between patients or independent infections from a common reservoir (Table S1, Figure 1).

### 3.5. K. Pneumoniae ST-Profile and AMR-Characteristics

All *bla*_CTX-M_ -positive *K. pneumoniae* (n = 17) were subjected to WGS for phylogenetic analyses, to examine potential clonal relatedness, and to verify PCR findings. Detailed information on the individual isolates is given in Supplementary Table S1 (ST1). Table 2 summarizes the ST profile and distribution of resistance determinants to clinically important classes of antibiotics for which we have phenotypic AST data: β-lactams, FQ, TMP, SUL, and AG.

WGS-analyses revealed 12 different STs with two isolates belonging to each of ST101, ST14, and ST340. We did not perform any sequence cluster analyses of *K. pneumoniae* isolates due to low numbers of identical STs. The presence of *bla*_CTX-M Group 1_ as detected by PCR was confirmed by WGS and *bla*_CTX-M-15_ was the only allelic variant. All 17 isolates were resistant to cefotaxime, ceftazidime, and cefuroxime. Other major β-lactamase encoding genes included *bla*_OXA-1_ (n = 11) and *bla*_TEM1-B_ (n = 9). SHV-variants except SHV-1 included SHV-28 (n = 4), SHV-11 (n = 4), SHV-187 (n = 1), and SHV-121 (n = 1), of which SHV-28 is associated with an ESBL-phenotype.

The dominant FQ-resistance determinants were *oqx*A (n = 9) and *qnrB1* (n = 4) as well as chromosomal mutations affecting the DNA gyrase (*gyrA*; n = 8). The corresponding isolates expressed resistance towards ciprofloxacin.

All *K. pneumoniae* isolates contained TMP- and SUL-resistance determinants. TMP-determinants included *dfr*_A12_, *dfr*_A14_, *dfr*_A17_, *dfr*_A27_, and *dfr*_A30_, of which *dfr*_A14_ (n = 13) was the dominant subtype. SUL-resistance genes were of subtypes *sul*_1_ and *sul*_2_, of which *sul*_2_ (n = 14) was dominant. All isolates expressed resistance to TMP-SUL.

The dominant AG-resistance determinants were *aac (3)-IIe* (n = 13), *aph (6)-Id* (n = 12), and *aph (3″)-Ib* (n = 11). All *K. pneumoniae* isolates that contained AG-resistance determinants expressed resistance to gentamicin.

### 3.6. Plasmid Incompatibility Groups

Sixteen plasmid incompatibility groups were identified from isolates of *K. pneumoniae*. IncF (n = 16/17; 94%) (IncFIB (n = 13/17; 76%), IncFII (n = 10/17; 71%), IncFIA (n = 9/17; 53%), and IncR (n = 12/17; 71%) groups were predominant. We did not perform any analyses of association between Inc-groups and pheno- or genotypic characteristics.

## 4. Discussion

This study adds to the knowledge on the molecular epidemiology of ESBL- and pAmpC-producing clinical isolates of *E. coli* and *K. pneumoniae* in Malawi. ESBLs were predominantly of CTX-M-15 and pAmpC were all CMY-2 type. CTX-M group 9 (*bla*_CTX-M-27 and -14_) were also detected. These observations are consistent with the global distribution of ESBL- [9,22] and pAmpC-subtypes (18) as well as previous studies from Sub-Saharan Africa [23,24] and Malawi [14,15,16]. CTX-M-15 has previously been shown to be the dominant ESBL type in invasive isolates of *E. coli* and *K. pneumoniae* from hospitalised adults and children in Malawi [14,15,16].

CMY-2 was the only detectable pAmpC type. This observation is consistent with recent findings in Ethiopia, where *bla*_CMY_ was the most frequent pAmpC in *E. coli* bacteremia isolates [25]. To our knowledge, there is no previous pAmpC-data for comparison in Malawi. Data from the neighbouring country Mozambique have shown the presence of both *bla*_CMY_, *bla*_DHA_, *bla*_FOX_, and *bla*_MOX_ in third-generation cephalosporin-resistant clinical isolates of *E. coli* [26]. These observations call for more surveillance of pAmpC to address the limited data for Malawi.

Isolates expressed an MDR-profile including resistance against FQ-, AG-, and/or TMP-SUL. These findings are in line with previous observations of MDR clinical isolates of ESBL-producing *E. coli* and *K. pneumoniae* in Malawi [14,15,16]. The dominant acquired FQ-resistance determinant for *E. coli* was *aac (6′)-Ib-cr*, while *qnrb1* and *oxqA* were most prevalent in *K. pneumoniae*. Both of the findings are comparable with previous observations in Sub-Saharan Africa [27] and Malawi [16]. However, *qnrS* in *K. pneumoniae* was previously reported in Malawi [16] but was not found in this study. These differences may be attributed to the fact that the corresponding specimens in this study were largely isolated from surgical specimens versus the study at Queen Elizabeth Central Hospital (QECH) Malawi which investigated *K. pneumoniae* isolated from blood cultures and rectal swabs [16]. To confer high-level FQ-resistance, additional chromosomal mutations are required. These chromosomal mutations were observed in our study, of which mutations in *gyr*A, *par* C, and *par*E were most prevalent for *E. coli* and mutations in *gyr*A only for *K. pneumoniae*. Mutations in *gyr*A in both *E. coli* and *K. pneumoniae* are comparable to recent findings in similar clinical isolates from Malawi [14,15,16], however *par* C and *par*E have not been previously reported in Malawi. Two of the 32 *E. coli* isolates that contained *gyrA* mutations expressed susceptibility to ciprofloxacin. These isolates contained only one codon mutation (S83L). Similar findings have been reported [14] whereby the presence of only one codon mutation did not confer phenotypic resistance to ciprofloxacin.

The dominant TMP-resistance genes were *dfr*_A17_ and *dfr*_A14_, in *E. coli* and *K. pneumoniae*, respectively. *Dfr*_A17_ has been previously described in *E. coli* in Malawi by Thega et al. (2021) [15] but, to our knowledge, this is the first report of dfrA14 in *K. pneumoniae* in Malawi. Musicha et al. [16] observed *dfr* in *K. pneumoniae*; however, they did not describe the subtype of *dfr* as such. Though potentially new to Malawi, these findings are common and concurring with WGS analyses conducted in Ghana on *K. pneumoniae* isolates resistant to third-generation cephalosporins [28]. *Sul_2_* was the most prevalent SUL-resistance determinant for both *E. coli* and *K. pneumoniae*, confirming the previous finding in Malawi [15,16].

ST410 and ST131 were the most prevalent STs for *E. coli*. These findings are in line with global ST distribution and also with previous studies in Malawi [14,15]. ST131 and ST410 are classified as high-risk MDR-clones of *E. coli* [29,30]. They are easily transmitted between patients, have the capability to colonise and persist in hosts, and may cause severe and recurrent infections [29]. Clonal dissemination of ST131 is in particular known to be associated with MDR [4]. MDR ST 410 has also shown clonal expansion over the past decade as evident from isolates of ST410 carrying the acquired carbapenamase gene *bla*_OXA-181_ being reported in Italy, China, and a small hospital outbreak in Denmark as described in [29]_._

ST410 isolates were prevalent in specimens from the surgical department (mainly pus) whereas ST131 was prevalent in specimens from the medical department (mainly blood culture and urine). This may indicate local transmission within the wards at the hospital, but the numbers are low. Similar to previous findings in Malawi [15], ST131 was associated with *bla*_CTX-M-15_ and *bla*_CTX-M-27_ in contrast to ST410 which was only associated with *bla*_CTX-M-15_. Clonal cluster analyses of the major STs of *E. coli* (ST 131, ST410 and ST617) in our study strongly indicate transmission between patients and/or to independent patients via a common reservoir. These observations support the need for strengthening infection-prevention measures, specifically the use of personal protective equipment (PPE) as well as the need to institute screening programs so that patients’ MDR isolates can be isolated to limit transmission

For *K. pneumoniae*, there was a high ST variation between the isolates. However, all the identified STs are considered global MDR clones [5]. These are clones that contribute disproportionately to the global disease burden and are among those clones that commonly cause hospital-acquired infections and outbreaks [5]. The identified STs in our study align with previous findings in Ghana (ST101) [28] and Malawi (ST14, ST340) [16,31,32]. ST101 has to our knowledge not previously been identified in Malawi.

Our findings and others [14,15,16] strongly suggest that globally distributed MDR-clones of *E. coli* and *K. pneumoniae* are already causing public health threats in hospitals in Malawi. Fortunately, in this study, we did not observe any carbapenem resistance, but the existing clones may acquire and display carbapenemase-encoding determinants [5]. Therefore, there is an urgent need to closely monitor the situation combined with stringent infection-control practices while carbapenem resistance is still low in Malawi.

A limitation of this study was that it only examined isolates from a single site and one region of the country. There may be variations in the type of ESBL and AmpC β -lactamases in different regions of the country. Many isolates were also lost in transit to the reference laboratory, thus reducing the number of isolates that could be sequenced. We were also unable to identify potential additional hospital-related sources of the MDR bacteria. ESBL-screening of hospital surfaces, medical devices/equipment, and patients could have added value to the findings of this study.

Infections with ESBLs and AmpC β-lactamase producing MDR Enterobacterales are of huge clinical importance. Their increasing rates drive the prescriptions of carbapenems which promotes the spread of potentially untreatable carbapenamase-producing Enterobacterales. Due to the limited availability of carbapenems at the study site, the current usage of this antibiotic class is limited. The high transmissibility of MDR-global clones calls for stringent infection-prevention practices and an urgent need for molecular and epidemiological studies to inform targeted containment strategies. The spread of these can have substantial effects on the healthcare systems of Malawi, where treatment options are already severely limited. Thus, such infections pose a financial burden on healthcare systems by increasing hospital stays and the cost of drugs which countries like Malawi may find difficult to sustain. Continuous surveillance and early detection are key for limiting their spread.

This study was able to provide insight into the molecular epidemiology of ESBL- and pAmpC-producing *E. coli* and *K. pneumoniae* in a tertiary hospital in Malawi. These findings add to the limited literature that exists for Malawi on the genomic characterisation of pathogens such as *E. coli* and *K. pneumoniae*. Together these data can be used to provide a baseline for purposes of tracking these MDR clones, and form a basis for policymakers to identify areas to target when addressing this major threat of antibiotic resistance.

## 5. Conclusions

MDR ESBL (CTX-M-15 type) and/or plasmid-mediated AmpC (CMY-type) producing *E. coli* and *K. pneumoniae* are prevalent in clinical specimens from KCH causing problems in recommended empirical treatment of bacterial infections. Most of the *E. coli* and *K. pneumoniae* isolates at KCH are representatives of high-risk MDR clones which are easily transmitted within the hospital. Strengthened diagnostic microbiology, close monitoring, and early detection for targeted infection-control measures are urgently needed.

## Figures and Tables

**Table 1:** ST-distribution and clinical important antimicrobial resistance determinants in *E. coli* strains (n = 37).

	PCR Result		β-Lactam Resistance						Trimethoprim Resistance					Sulphonamide Resistance		Quinolone Mutations			Aminoglycoside
ST	CTX-M +	pAmpC +	CTX-M-15 (n = 30)	CTX-M-27 (n = 6)	CTX-M-14 (n = 1)	CMY-2 (n = 15)	TEM-1 (n = 32)	OXA-1 (n = 23)	dfrA1 (n = 2)	dfrA8 (n = 1)	dfrA12 (n = 2)	dfrA14 (n = 1)	dfrA17 (n = 31)	sul 1 (n = 33)	sul 2 (n = 35)	gyrA (n = 32)	parC (n = 29)	parE (n = 31)	(n = 37)
ST410 (n = 16)	16	1/5	16	-	-	15	15	14	-	-	-	-	16	16	15	16	15	15	16
ST131 (n = 7)	7	-	2	5		-	4	-	-	-	-	-	7	7	7	7	4	7	7
ST617 (n = 6)	6	-	6	-	-	-	6	6			1		5	6	6	5	6	6	6
ST155 (n = 1)	1	-	1	-	-	-	1	-	-	-	-	-	-	-	-	1	-	-	1
ST48 (n = 1)	1	-	1	-	-	-	1	-	-	-	1	-	-	1	1	1	-	-	1
ST6332 (n = 1)	1	-	1	-	-	-	1	1	-	-	-	1	-	-	1	1	1	1	1
ST354 (n = 1)	1	-	-	1	-	-	1	-	-	-	-	-	1	1	1	1	1	1	1
ST5824 (n = 1)	1	-	-	-	1	-	-	-	1	-	-	-	-	-	1	-	-	-	1
ST38 (n = 1)	1	-	1	-	-	-	1	-	1	-	-	-	-	-	1	-	-	-	1
ST44 (n = 1)	1	-	1	-	-	-	1	1	-	1	-	-	1	1	1		1	-	1
ST648 (n = 1)	1	-	1	-	-	-	1	1	-	-	-	-	1	1	1	1	1	1	1

**Table 2:** ST-distribution and clinical important antimicrobial resistance determinants in K. pneumoniae strains (n = 17).

ST	PCR Result	β-Lactam Resistance					Trimethoprim Resistance						Sulphonamide Resistance		Quinolone Mutations		Amino Glycoside
	CTX-M (n = 17)	CTX-M-15 (n = 17)	SHV (n = 16)	TEM (n = 14)	OXA (n = 11)	blaSCO-1 (n = 1)	dfrA1 (n = 1)	dfrA12 (n = 1)	dfrA14 (n = 12)	dfrA17 (n = 1)	dfrA27 (n = 1)	dfrA30 (n = 1)	Sul1 (n = 6)	Sul2 (n = 14)	gyrA (n = 8)	qnrB1 (n = 4)	(n = 17)
ST101 (n = 2)	2	2	2	-	2	-			2					2	2		2
ST1047 (n = 1)	1	1	1	1	1	-			1					1			1
ST14 (n = 2)	2	2	2	2	2	-			1			1		2		1	2
ST15 (n = 1)	1	1	1	1	1	-			1				1	1	1		1
ST1552 (n = 1)	1	1	1	1	-	-	1		1				1	1		1	1
ST231 (n = 1)	1	1	1	1	1	-			1						1		1
ST29 (n = 1)	1	1	1	1	1	-								1		1	1
ST307 (n = 1)	1	1	1	1	-	-			1				1	1			1
ST340 (n = 2)	2	2	2	2	1	-			2				1	1	2		2
ST48 (n = 1)	1	1	1	1	1	-		1					1	1	1		1
ST607 (n = 1)	1	1	1	-	-	1					1			1			1
ST874 (n = 1)	1	1	1	1	1	-			1				1	1	1		1
UNKNOWN (n = 2)	2	2	1	1	1	-			1	1				1		1	2

## Data Availability

The datasets used and/or analysed in this study are available from the corresponding author on reasonable request.

## Supplementary Materials

The following supporting information can be downloaded at https://www.mdpi.com/article/10.3390/tropicalmed7090245/s1. Table S1: v2 26.6.22.
