# Supplementary material for: Molecular Epidemiological Characterisation of ESBL- and Plasmid-Mediated AmpC-Producing Escherichia coli and Klebsiella pneumoniae at Kamuzu Central Hospital, Lilongwe, Malawi
Source: Trop Med Infect Dis. 2022 Sep 14;7(9):245. doi: 10.3390/tropicalmed7090245 (PMC9501462; doi:10.3390/tropicalmed7090245)
Supplement: Supplementary file 1 [file tropicalmed-07-00245-s001.zip › tropicalmed-1876747-supplementary.pdf]

RID	KID	ward	specimen type	Species	MLST	No. contigs
P30_79	10A	surgical	pus	Escherichia_coli	ST-617	237
P30_80	38	surgical	pus	Escherichia_coli	ST-410	117
P30_81	39	surgical	pus	Escherichia_coli	ST-617	404
P31_01	64	surgical	pus	Escherichia_coli	ST-410	186
P31_04	89b	surgical	pus	Escherichia_coli	ST-410	148
P31_05	90	surgical	urine	Escherichia_coli	ST-410	199
P31_06	93 b	medical	urine	Escherichia_coli	ST-44	269
P31_07	99	surgical	pus	Escherichia_coli	ST-155	116
P31_08	100	surgical	pus	Escherichia_coli	ST-410	153
P31_10	143a	surgical	urine	Escherichia_coli	ST-6332	193
P31_11	145	surgical	pus	Escherichia_coli	ST-354	138
P31_12	146	surgical	pus	Escherichia_coli	ST-131	163
P31_13	150a	surgical	pus	Escherichia_coli	ST-410	161
P31_14	162	medical	urine	Escherichia_coli	ST-410	142
P31_15	168	medical	urine	Escherichia_coli	ST-410	194
P31_16	193	surgical	pus	Escherichia_coli	ST-131	139
P31_17	194	surgical	pus	Escherichia_coli	ST-48	291
P31_18	208	surgical	Labelled 215 A.	Escherichia_coli	ST-410	115
P31_20	219	medical	urine	Escherichia_coli	ST-410	121
P31_22	222	medical	urine	Escherichia_coli	ST-410	155
P31_23	226	medical	blood culture	Escherichia_coli	ST-410	126
P31_25	249B	surgical	pus	Escherichia_coli	ST-38	183
P31_26	277	surgical	pus	Escherichia_coli	ST-410	127
P31_27	348A	surgical	urine	Escherichia_coli	ST-131	158
P31_30	405	surgical	pus	Escherichia_coli	ST-648	188
P31_31	443	medical	blood culture	Escherichia_coli	ST-131	225
P31_33	502A	surgical	pus	Escherichia_coli	ST-131	205
P31_34	520	obs&gynae	pus	Escherichia_coli	ST-5824	243
P31_36	583	surgical		Escherichia_coli	ST-131	136
P31_37	585	surgical	pus	Escherichia_coli	ST-410	142
P31_39	604	surgical	pus	Escherichia_coli	ST-410	120
P31_40	615	surgical	pus	Escherichia_coli	ST-617	220
P31_43	634B	surgical	pus	Escherichia_coli	ST-617	191
P31_44	639	surgical	pus	Escherichia_coli	ST-410	121
P31_47	266	medical	blood culture	Escherichia_coli	ST-131	162
P31_51	348B	surgical	urine	Escherichia_coli	ST-617	174
P31_58	615	surgical	pus	Escherichia_coli	ST-617	238

Length	Coverage	Aminoglycosides
5053406	128.435	aph(6)-Id(100.00,100.00) | aph(3'')-Ib(100.00,100.
4821435	153.572	aac(3)-IId(100.00,100.00) | aph(6)-Id(100.00,100.
5146341	144.84	aac(3)-IId(100.00,100.00) | aadA5(100.00,100.00)
4856558	150.064	aadA5(100.00,100.00) | aph(6)-Id(100.00,100.00)
4906331	156.292	aadA5(100.00,100.00) | aac(3)-IId(100.00,100.00)
4909005	149.481	aadA5(100.00,100.00) | aph(6)-Id(100.00,100.00)
4925961	179.44	aadA5(100.00,100.00) | aph(6)-Id(100.00,100.00)
4877359	120.412	aac(3)-IId(100.00,100.00)
4901033	149.398	aadA5(100.00,100.00) | aac(3)-IId(100.00,100.00)
4900455	155.752	aph(6)-Id(100.00,100.00) | aph(3'')-Ib(100.00,100.
5040849	114.608	aadA5(100.00,100.00) | aph(6)-Id(100.00,100.00)
5102809	120.845	aadA5(100.00,100.00) | aph(3'')-Ib(100.00,100.00
4896957	141.706	aadA5(100.00,100.00) | aac(3)-IId(100.00,100.00)
4909249	112.607	aadA5(100.00,100.00) | aac(3)-IId(100.00,100.00)
4912714	241.745	aadA5(100.00,100.00) | aph(6)-Id(100.00,100.00)
5154208	112.871	aadA5(100.00,100.00) | aph(6)-Id(100.00,100.00)
4803866	115.526	aph(6)-Id(100.00,100.00) | aph(3'')-Ib(100.00,100.
4848605	146.061	aadA5(100.00,100.00) | aph(6)-Id(100.00,100.00)
4889860	135.075	aadA5(100.00,100.00) | aac(3)-IId(100.00,100.00)
4818732	143.477	aadA5(100.00,100.00) | aph(6)-Id(100.00,100.00)
4818990	129.043	aadA5(100.00,100.00) | aac(3)-IId(100.00,100.00)
5403751	55.7029	aadA1(100.00,100.00) | aph(6)-Id(100.00,100.00)
4830344	150.564	aac(3)-IId(100.00,100.00) | aadA5(100.00,100.00)
5087754	106.167	aadA5(100.00,100.00) | aph(6)-Id(100.00,100.00)
5287481	145.377	aadA5(100.00,100.00) | aph(3'')-Ib(100.00,100.00
5233250	118.84	aadA5(100.00,100.00) | aph(3'')-Ib(100.00,100.00
5044830	116.055	aadA5(100.00,100.00) | aph(6)-Id(100.00,100.00)
4989743	116.139	aph(6)-Id(100.00,100.00) | aadA1(100.00,100.00)
5079574	121.85	aadA5(100.00,100.00) | aph(6)-Id(100.00,100.00)
4813475	113.754	aadA5(100.00,100.00) | aac(3)-IId(100.00,100.00)
4825829	116.221	aadA5(100.00,100.00) | aph(6)-Id(100.00,100.00)
5213707	110.393	aac(3)-IId(100.00,100.00) | aadA5(100.00,100.00)
5060787	255.787	aac(3)-IId(100.00,100.00) | aadA5(100.00,100.00)
4801908	138.334	aadA5(100.00,100.00) | aph(3'')-Ib(100.00,100.00
5092957	110.788	aadA5(100.00,100.00) | aph(3'')-Ib(100.00,100.00
4938514	79.7426	aadA2(100.00,100.00) | aph(3'')-Ib(100.00,100.00
5214881	116.321	aph(6)-Id(100.00,100.00) | aph(3'')-Ib(100.00,100.

Aminoglycosides/quinolones	Betalactams	Phenicols
aac(6')-Ib-cr5(100.00,100.00)	blaCTX-M-15(100.00,100.00) | blaOX	catA1(100.00,100.00)
aac(6')-Ib-cr5(100.00,100.00)	blaCMY-2(100.00,100.00) | blaTEM-1	nil
aac(6')-Ib-cr5(100.00,100.00)	blaCTX-M-15(100.00,100.00) | blaOX	catA1(100.00,100.00)
aac(6')-Ib-cr5(100.00,100.00)	blaOXA-1(100.00,100.00) | blaCTX-M	nil
aac(6')-Ib-cr5(100.00,100.00)	blaCMY-2(100.00,100.00) | blaTEM-1	nil
aac(6')-Ib-cr5(100.00,100.00)	blaCMY-2(100.00,100.00) | blaTEM-1	nil
aac(6')-Ib-cr5(100.00,100.00)	blaCTX-M-15(100.00,100.00) | blaTE	nil
nil	blaCTX-M-15(100.00,100.00) | blaTE	nil
aac(6')-Ib-cr5(100.00,100.00)	blaCMY-2(100.00,100.00) | blaTEM-1	nil
aac(6')-Ib-cr5(100.00,100.00)	blaCTX-M-15(100.00,100.00) | blaOX	nil
nil	blaCTX-M-27(100.00,100.00) | blaTE	nil
nil	blaTEM-1(100.00,100.00) | blaCTX-M	nil
aac(6')-Ib-cr5(100.00,100.00)	blaCMY-2(100.00,100.00) | blaTEM-1	nil
aac(6')-Ib-cr5(100.00,100.00)	blaCMY-2(100.00,100.00) | blaTEM-1	nil
aac(6')-Ib-cr5(100.00,100.00)	blaCMY-2(100.00,100.00) | blaCTX-M	nil
nil	blaCTX-M-27(100.00,100.00)	nil
nil	blaCTX-M-15(100.00,100.00) | blaTE	catA1(100.00,100.00)
aac(6')-Ib-cr5(100.00,100.00)	blaCMY-2(100.00,100.00) | blaTEM-1	nil
aac(6')-Ib-cr5(100.00,100.00)	blaCMY-2(100.00,100.00) | blaTEM-1	nil
aac(6')-Ib-cr5(100.00,100.00)	blaCMY-2(100.00,100.00) | blaTEM-1	nil
aac(6')-Ib-cr5(100.00,100.00)	blaCMY-2(100.00,100.00) | blaTEM-1	nil
nil	blaCTX-M-15(100.00,100.00) | blaTE	nil
aac(6')-Ib-cr5(100.00,100.00)	blaCMY-2(100.00,100.00) | blaTEM-1	nil
nil	blaCTX-M-27(100.00,100.00) | blaTE	nil
aac(6')-Ib-cr5(100.00,100.00)	blaTEM-1(100.00,100.00) | blaCTX-M	catA1(100.00,100.00)
nil	blaCTX-M-27(100.00,100.00)	nil
nil	blaTEM-1(100.00,100.00) | blaCTX-M	nil
nil	blaCTX-M-14(100.00,100.00)	nil
nil	blaCTX-M-27(100.00,100.00)	nil
aac(6')-Ib-cr5(100.00,100.00)	blaCMY-2(100.00,100.00) | blaTEM-1	nil
aac(6')-Ib-cr5(100.00,100.00)	blaCMY-2(100.00,100.00) | blaTEM-1	nil
aac(6')-Ib-cr5(100.00,100.00)	blaOXA-1(100.00,100.00) | blaTEM-1	catA1(100.00,100.00)
aac(6')-Ib-cr5(100.00,100.00)	blaCTX-M-15(100.00,100.00) | blaOX	catA1(100.00,100.00)
aac(6')-Ib-cr5(100.00,100.00)	blaCMY-2(100.00,100.00) | blaCTX-M	nil
nil	blaCTX-M-15(100.00,100.00) | blaTE	nil
aac(6')-Ib-cr5(100.00,100.00)	blaTEM-1(100.00,100.00) | blaCTX-M	nil
aac(6')-Ib-cr5(100.00,100.00)	blaCTX-M-15(100.00,100.00) | blaOX	catA1(100.00,100.00)

Quarternary ammonium cc	Macrolides	Streptothricin
qacEdelta1(100.00,100.00)	mph(A)(100.00,100.00)	nil
qacEdelta1(100.00,100.00)	mph(A)(100.00,100.00)	nil
qacEdelta1(100.00,100.00)	mph(A)(100.00,100.00)	nil
qacEdelta1(100.00,100.00)	mph(A)(100.00,100.00)	nil
qacEdelta1(100.00,100.00)	mph(A)(100.00,100.00)	nil
qacEdelta1(100.00,100.00)	mph(A)(100.00,100.00)	nil
qacEdelta1(100.00,100.00)	mph(A)(100.00,99.67)	nil
nil	nil	nil
qacEdelta1(100.00,100.00)	mph(A)(100.00,100.00)	nil
nil	mph(A)(100.00,100.00)	nil
qacEdelta1(100.00,100.00)	mph(A)(100.00,100.00) | erm(	nil
qacEdelta1(100.00,100.00)	mph(A)(100.00,100.00)	nil
qacEdelta1(100.00,100.00)	mph(A)(100.00,100.00)	nil
qacEdelta1(100.00,100.00)	mph(A)(100.00,100.00)	nil
qacEdelta1(100.00,100.00)	mph(A)(100.00,100.00)	nil
qacEdelta1(100.00,100.00)	mph(A)(100.00,99.67)	nil
qacEdelta1(100.00,100.00)	mph(A)(100.00,100.00)	nil
qacEdelta1(100.00,100.00)	mph(A)(100.00,100.00)	nil
qacEdelta1(100.00,100.00)	mph(A)(100.00,100.00)	nil
qacEdelta1(100.00,100.00)	mph(A)(100.00,100.00)	nil
qacEdelta1(100.00,100.00)	mph(A)(100.00,100.00)	nil
nil	nil	sat2(100.00,100.00)
qacEdelta1(100.00,100.00)	mph(A)(100.00,100.00)	nil
qacEdelta1(100.00,100.00)	mph(A)(100.00,100.00)	nil
qacEdelta1(100.00,100.00)	mph(A)(100.00,100.00)	nil
qacEdelta1(100.00,100.00)	mph(A)(100.00,100.00)	nil
qacEdelta1(100.00,100.00)	mph(A)(100.00,100.00)	nil
nil	nil	sat2(100.00,100.00)
qacEdelta1(100.00,100.00)	mph(A)(100.00,100.00)	nil
qacEdelta1(100.00,100.00)	mph(A)(100.00,100.00)	nil
qacEdelta1(100.00,100.00)	mph(A)(100.00,100.00)	nil
qacEdelta1(100.00,100.00)	mph(A)(100.00,100.00)	nil
qacEdelta1(100.00,100.00)	mph(A)(100.00,100.00)	nil
qacEdelta1(100.00,100.00)	mph(A)(100.00,100.00)	nil
qacEdelta1(100.00,100.00)	mph(A)(100.00,100.00)	nil
qacEdelta1(100.00,100.00)	mph(A)(100.00,100.00)	nil
qacEdelta1(100.00,100.00)	mph(A)(100.00,100.00)	nil

Sulfonamider	Tetrasykliner
sul2(100.00,100.00) | sul1(100.00,100.00)	tet(B)(100.00,100.00)
sul2(100.00,100.00) | sul1(100.00,100.00)	tet(B)(100.00,100.00)
sul1(100.00,100.00) | sul2(100.00,100.00)	tet(B)(100.00,100.00)
sul1(100.00,100.00) | sul2(100.00,100.00)	tet(A)(100.00,100.00)
sul1(100.00,100.00) | sul2(100.00,100.00)	tet(B)(100.00,100.00)
sul1(100.00,100.00) | sul2(100.00,100.00)	tet(B)(100.00,100.00)
sul1(100.00,100.00) | sul2(100.00,100.00)	tet(B)(100.00,100.00)
nil	nil
sul1(100.00,100.00) | sul2(100.00,100.00)	tet(B)(100.00,100.00)
sul2(100.00,100.00)	tet(A)(100.00,100.00)
sul1(100.00,100.00) | sul2(100.00,100.00)	tet(B)(100.00,100.00)
sul1(100.00,100.00) | sul2(100.00,100.00)	tet(A)(100.00,100.00)
sul1(100.00,100.00) | sul2(100.00,100.00)	tet(B)(100.00,100.00)
sul1(100.00,100.00) | sul2(100.00,100.00)	tet(B)(100.00,100.00)
sul1(100.00,100.00) | sul2(100.00,100.00)	tet(B)(100.00,100.00)
sul1(100.00,100.00) | sul2(100.00,100.00)	tet(A)(100.00,100.00)
sul2(100.00,100.00) | sul1(100.00,100.00)	tet(B)(100.00,100.00)
sul1(100.00,100.00) | sul2(100.00,100.00)	tet(B)(100.00,100.00)
sul1(100.00,100.00)	tet(B)(100.00,100.00)
sul1(100.00,100.00) | sul2(100.00,100.00)	tet(B)(100.00,100.00)
sul1(100.00,100.00) | sul2(100.00,100.00)	tet(B)(100.00,100.00)
sul2(100.00,100.00)	tet(D)(100.00,100.00)
sul1(100.00,100.00) | sul2(100.00,100.00)	tet(B)(100.00,100.00)
sul1(100.00,100.00) | sul2(100.00,100.00)	tet(A)(100.00,100.00)
sul1(100.00,100.00) | sul2(100.00,100.00)	tet(B)(100.00,100.00)
sul1(100.00,100.00) | sul2(100.00,100.00)	tet(A)(100.00,100.00)
sul1(100.00,100.00) | sul2(100.00,100.00)	tet(A)(100.00,100.00)
sul2(100.00,100.00)	tet(B)(100.00,100.00)
sul1(100.00,99.64) | sul2(100.00,100.00)	tet(A)(100.00,100.00)
sul1(100.00,100.00) | sul2(100.00,100.00)	tet(B)(100.00,100.00)
sul1(100.00,100.00) | sul2(100.00,100.00)	tet(B)(100.00,100.00)
sul1(100.00,100.00) | sul2(100.00,100.00)	tet(B)(100.00,100.00)
sul1(100.00,100.00) | sul2(100.00,100.00)	tet(B)(100.00,100.00)
sul1(100.00,100.00) | sul2(100.00,100.00)	tet(B)(100.00,100.00)
sul1(100.00,100.00) | sul2(100.00,100.00)	tet(A)(100.00,100.00)
sul1(100.00,100.00) | sul2(100.00,100.00)	tet(B)(100.00,100.00)
sul2(100.00,100.00) | sul1(100.00,100.00)	tet(B)(100.00,100.00)

Trimetoprim	Fosfomycin	Fosmidomycin
dfrA17(100.00,100.00)	nil	nil
dfrA17(94.27,100.00)	nil	nil
dfrA17(100.00,100.00)	nil	nil
dfrA17(100.00,100.00)	nil	nil
dfrA17(100.00,100.00)	nil	nil
dfrA17(100.00,100.00)	nil	nil
dfrA17(100.00,100.00) | dfrA8(100.00,100.00)	nil	nil
nil	nil	nil
dfrA17(100.00,100.00)	nil	nil
dfrA14(100.00,100.00)	nil	nil
dfrA17(100.00,100.00)	uhpT_E350Q	cyaA_S352T(100.00,99.29)
dfrA17(100.00,100.00)	uhpT_E350Q	nil
dfrA17(100.00,100.00)	nil	nil
dfrA17(100.00,100.00)	nil	nil
dfrA17(100.00,100.00)	nil	nil
dfrA17(100.00,100.00)	uhpT_E350Q	nil
dfrA12(100.00,100.00)	nil	nil
dfrA17(100.00,100.00)	nil	nil
dfrA17(100.00,100.00)	nil	nil
dfrA17(100.00,100.00)	nil	nil
dfrA17(100.00,100.00)	nil	nil
dfrA1(100.00,100.00)	nil	nil
dfrA17(100.00,100.00)	nil	nil
dfrA17(92.99,100.00)	uhpT_E350Q	nil
dfrA17(100.00,100.00)	nil	cyaA_S352T(100.00,99.29)
dfrA17(100.00,100.00)	ptsI_V25I(10(	nil
dfrA17(100.00,100.00)	uhpT_E350Q	nil
dfrA1(100.00,100.00)	nil	nil
dfrA17(100.00,100.00)	ptsI_V25I(10(	nil
dfrA17(100.00,100.00)	nil	nil
dfrA17(100.00,100.00)	nil	nil
dfrA17(100.00,100.00)	nil	nil
dfrA17(100.00,100.00)	nil	nil
dfrA17(100.00,100.00)	nil	nil
dfrA17(100.00,100.00)	uhpT_E350Q	nil
dfrA12(100.00,100.00)	nil	nil
dfrA17(100.00,100.00)	nil	nil

Quinolones_mutation

parE_S458A(100.00,99.68) | parC_S80I(100.00,99.87) | [illegible]
parC_S80I(100.00,99.87) | parE_S458A(100.00,99.68) | [illegible]
parC_S80I(100.00,99.87) | parE_S458A(100.00,99.68) | [illegible]
gyrA_D87N(99.66,98.97) | gyrA_S83L(99.66,98.97) | par[illegible]
parC_S80I(100.00,99.87) | parE_S458A(100.00,99.68) | [illegible]
parC_S80I(100.00,99.87) | parE_S458A(100.00,99.68) | [illegible]
nil
nil
parE_S458A(100.00,99.68) | parC_S80I(100.00,99.87) | [illegible]
parC_S80I(100.00,99.87) | parE_S458A(100.00,99.68) | [illegible]
gyrA_D87N(99.66,98.97) | gyrA_S83L(99.66,98.97) | par[illegible]
parE_I529L(100.00,99.37) | parE_S458A(100.00,99.37) | [illegible]
parC_S80I(100.00,99.87) | parE_S458A(100.00,99.68) | [illegible]
parC_S80I(100.00,99.87) | parE_S458A(100.00,99.68) | [illegible]
parC_S80I(100.00,99.87) | parE_S458A(100.00,99.68) | [illegible]
gyrA_D87N(99.66,98.86) | gyrA_S83L(99.66,98.86) | par[illegible]
gyrA_S83A(99.66,99.09)
parC_S80I(100.00,99.87) | parE_S458A(100.00,99.68) | [illegible]
gyrA_D87N(99.66,98.97) | gyrA_S83L(99.66,98.97) | par[illegible]
parE_S458A(100.00,99.68) | parC_S80I(100.00,99.87) | [illegible]
parC_S80I(100.00,99.87) | parE_S458A(100.00,99.68) | [illegible]
nil
gyrA_D87N(99.66,98.97) | gyrA_S83L(99.66,98.97) | par[illegible]
gyrA_D87N(99.66,98.86) | gyrA_S83L(99.66,98.86) | par[illegible]
gyrA_D87N(99.66,98.74) | gyrA_S83L(99.66,98.74) | par[illegible]
parC_E84V(100.00,99.20) | parC_S80I(100.00,99.20) | p[illegible]
parE_E460D(100.00,99.68) | parC_S80I(100.00,99.60) | [illegible]
nil
gyrA_D87N(99.66,98.86) | gyrA_S83L(99.66,98.86) | par[illegible]
gyrA_D87N(99.66,98.97) | gyrA_S83L(99.66,98.97) | par[illegible]
parC_S80I(100.00,99.87) | parE_S458A(100.00,99.68) | [illegible]
gyrA_D87N(99.66,98.86) | gyrA_S83L(99.66,98.86) | par[illegible]
nil
parC_S80I(100.00,99.87) | parE_S458A(100.00,99.68) | [illegible]
gyrA_S83L(99.66,98.86) | parE_I529L(100.00,99.52)
parE_S458A(100.00,99.68) | parC_S80I(100.00,99.87) | [illegible]
parC_S80I(100.00,99.87) | parE_S458A(100.00,99.68) | [illegible]

Plasmids

Col(pHAD28)_1_KU674895 | FIA(pBK30683)_1_KF954760 | IncFIA_1_AP001918 | IncFIB(AP001
FIA(pBK30683)_1_KF954760 | IncFIA_1_AP001918 | IncFIB(AP001918)_1_AP001918 | IncFII(pA
Col(pHAD28)_1_KU674895 | FIA(pBK30683)_1_KF954760 | IncFIA_1_AP001918 | IncFIB(AP001
Col(pHAD28)_1_KU674895 | Col156_1_NC_009781 | FIA(pBK30683)_1_KF954760 | IncFIA_1_A
Col(BS512)_1_NC_010656 | Col(MG828)_1_NC_008486 | FIA(pBK30683)_1_KF954760 | IncFIA_
Col(BS512)_1_NC_010656 | Col(MG828)_1_NC_008486 | FIA(pBK30683)_1_KF954760 | IncFIA_
Col(BS512)_1_NC_010656 | Col(MG828)_1_NC_008486 | Col(pHAD28)_1_KU674895 | Col440I_
Col(pHAD28)_1_KU674895 | FIA(pBK30683)_1_KF954760 | IncFIA_1_AP001918 | IncFIB(S)_1_
Col(BS512)_1_NC_010656 | Col(MG828)_1_NC_008486 | FIA(pBK30683)_1_KF954760 | IncFIA_
FIA(pBK30683)_1_KF954760 | IncFIA_1_AP001918 | IncFIB(AP001918)_1_AP001918 | IncFIC(F
FIA(pBK30683)_1_KF954760 | IncFIA_1_AP001918 | IncFIB(S)_1_FN432031 | IncFIB(pB171)_1_
Col156_1_NC_009781 | FIA(pBK30683)_1_KF954760 | IncFIA_1_AP001918 | IncFIB(AP001918)
Col(BS512)_1_NC_010656 | Col(MG828)_1_NC_008486 | FIA(pBK30683)_1_KF954760 | IncFIA_
Col(BS512)_1_NC_010656 | Col(MG828)_1_NC_008486 | FIA(pBK30683)_1_KF954760 | IncFIA_
Col(BS512)_1_NC_010656 | Col(MG828)_1_NC_008486 | FIA(pBK30683)_1_KF954760 | IncFIA_
Col156_1_NC_009781 | Col(MG828)_1_NC_008486 | FIA(pBK30683)_1_KF954760 | IncFIA_1_A
Col(BS512)_1_NC_010656 | Col(MG828)_1_NC_008486 | Col(pHAD28)_1_KU674895 | IncFIB(A
FIA(pBK30683)_1_KF954760 | IncFIA_1_AP001918 | IncFIB(AP001918)_1_AP001918 | IncFII_1_
Col(BS512)_1_NC_010656 | Col(MG828)_1_NC_008486 | FIA(pBK30683)_1_KF954760 | IncFIA_
Col(BS512)_1_NC_010656 | FIA(pBK30683)_1_KF954760 | IncFIA_1_AP001918 | IncFIB(AP001
Col(BS512)_1_NC_010656 | FIA(pBK30683)_1_KF954760 | IncFIA_1_AP001918 | IncFIB(AP001
IncFIB(AP001918)_1_AP001918 | IncFII(29)_1_CP003035 | IncFII(pAMA1167-NDM-5)_1_CP024
Col(BS512)_1_NC_010656 | FIA(pBK30683)_1_KF954760 | IncFIA_1_AP001918 | IncFIB(AP001
Col156_1_NC_009781 | Col(MG828)_1_NC_008486 | ColRNAI_1_DQ298019 | FIA(pBK30683)_1
Col(BS512)_1_NC_010656 | Col(pHAD28)_1_KU674895 | FIA(pBK30683)_1_KF954760 | IncFIA_
Col156_1_NC_009781 | Col(MG828)_1_NC_008486 | FIA(pBK30683)_1_KF954760 | IncFIA_1_A
Col156_1_NC_009781 | ColRNAI_1_DQ298019 | FIA(pBK30683)_1_KF954760 | IncFIA_1_AP00
Col156_1_NC_009781 | Col(BS512)_1_NC_010656 | Col(pHAD28)_1_KU674895 | IncFIB(AP001
Col156_1_NC_009781 | FIA(pBK30683)_1_KF954760 | IncFIA_1_AP001918 | IncFIB(AP001918)
Col(BS512)_1_NC_010656 | FIA(pBK30683)_1_KF954760 | IncFIA_1_AP001918 | IncFIB(AP001
Col(BS512)_1_NC_010656 | FIA(pBK30683)_1_KF954760 | IncFIA_1_AP001918 | IncFIB(AP001
Col(pHAD28)_1_KU674895 | FIA(pBK30683)_1_KF954760 | IncFIA_1_AP001918 | IncFIB(AP001
Col(pHAD28)_1_KU674895 | FIA(pBK30683)_1_KF954760 | IncFIA_1_AP001918 | IncFIB(AP001
FIA(pBK30683)_1_KF954760 | IncFIA_1_AP001918 | IncFIB(AP001918)_1_AP001918 | IncFIC(F
Col156_1_NC_009781 | IncFIB(AP001918)_1_AP001918 | IncFIC(FII)_1_AP001918 | IncFII(29)_
Col(pHAD28)_1_KU674895 | FIA(pBK30683)_1_KF954760 | IncFIA_1_AP001918 | IncFIB(AP001
Col(pHAD28)_1_KU674895 | FIA(pBK30683)_1_KF954760 | IncFIA_1_AP001918 | IncFIB(AP001

≥20/<17		≥19/<19		≥19/<19	
**CTX**	**CTX**	**FOX**	**FOX**	**CXM**	**CXM**
6	R	18	R	6	R
6	R	6	R	6	R
6	R	20	S	6	R
6	R	21	S	6	R
6	R	6	R	6	R
6	R	6	R	6	R
6	R	21	S	6	R
6	R	24	S	6	R
6	R	6	R	6	R
10	R	23	S	6	R
6	R	18	R	6	R
6	R	21	S	6	R
6	R	6	R	6	R
6	R	6	R	6	R
6	R	6	R	6	R
6	R	19	S	6	R
6	R	26	S	6	R
6	R	6	R	6	R
6	R	6	R	6	R
6	R	6	R	6	R
6	R	6	R	6	R
6	R	23	S	6	R
6	R	6	R	6	R
6	R	23	S	6	R
6	R	20	S	6	R
6	R	22	S	6	R
8	R	24	S	6	R
6	R	23	S	6	R
6	R	22	S	6	R
6	R	6	R	6	R
6	R	6	R	6	R
6	R	20	S	6	R
6	R	21	S	6	R
8	R	6	R	6	R
6	R	22	S	6	R
6	R	6	R	6	R
6	R	21	S	6	R

≥14/<11		≥17/<14		≥26/<24		≥22/<16
**SXT**	**SXT**	**CN10**	**CN10**	**CIP**	**CIP**	**MEM**
6	R	6	R	6	R	29
6	R	8	R	6	R	27
6	R	6	R	6	R	29
6	R	19	S	6	R	29
6	R	7	R	6	R	27
6	R	8	R	6	R	26
6	R	8	R	6	R	29
25	S	7	R	27	S	28
6	R	6	R	6	R	28
6	R	7	R	6	R	30
6	R	20	S	6	R	32
6	R	6	R	20	S	29
6	R	8	R	6	R	28
6	R	8	R	6	R	27
6	R	8	R	6	R	28
6	R	20	S	6	R	31
11	R	10	R	26	S	28
6	R	7	R	6	R	29
6	R	8	R	6	R	25
6	R	7	R	6	R	28
6	R	9	R	6	R	30
6	R	22	S	24	S	30
6	R	8	R	6	R	28
6	R	18	S	6	R	31
6	R	6	R	6	R	28
6	R	20	S	6	R	31
6	R	19	S	6	R	31
6	R	6	R	6	R	29
6	R	19	S	6	R	28
6	R	8	R	6	R	28
6	R	8	R	6	R	26
6	R	8	R	6	R	28
6	R	8	R	6	R	30
6	R	6	R	6	R	27
6	R	19	S	24	S	30
6	R	6	R	6	R	30
6	R	8	R	6	R	30

	≥20/<17		≥26/<21		≥22/<19	
**MEM**	**TZP**	**TZP**	**ATM**	**ATM**	**CAZ**	**CAZ**
S	15	R	8	R	8	R
S	10	R	6	R	6	R
S	10	R	7	R	7	R
S	18	I	10	R	9	R
S	8	R	6	R	6	R
S	8	R	6	R	6	R
S	16	R	10	R	10	R
S	21	S	14	R	13	R
S	10	R	6	R	6	R
S	16	R	10	R	10	R
S	23	S	16	R	16	R
S	21	S	14	R	15	R
S	8	R	6	R	6	R
S	9	R	6	R	6	R
S	9	R	6	R	6	R
S	21	S	15	R	13	R
S	23	S	19	R	18	R
S	9	R	6	R	6	R
S	8	R	6	R	6	R
S	8	R	6	R	6	R
S	9	R	6	R	6	R
S	22	S	11	R	11	R
S	9	R	6	R	6	R
S	23	S	19	R	17	R
S	18	I	11	R	12	R
S	23	S	20	R	17	R
S	22	S	19	R	17	R
S	20	S	14	R	13	R
S	22	S	18	R	11	R
S	10	R	6	R	6	R
S	9	R	6	R	6	R
S	18	I	12	R	12	R
S	18	I	13	R	13	R
S	14	R	6	R	6	R
S	22	S	17	R	18	R
S	6	R	6	R	6	R
S	17	I	12	R	12	R
